# Acetylation at lysine 71 inactivates superoxide dismutase 1 and sensitizes cancer cells to genotoxic agents

**DOI:** 10.18632/oncotarget.3987

**Published:** 2015-05-04

**Authors:** Chenchu Lin, Hanlin Zeng, Junyan Lu, Zuoquan Xie, Wenyi Sun, Cheng Luo, Jian Ding, Shengtao Yuan, Meiyu Geng, Min Huang

**Affiliations:** ^1^ Division of Anti-Tumor Pharmacology, State Key Laboratory of Drug Research, Shanghai Institute of Materia Medica, Chinese Academy of Sciences, Shanghai, China; ^2^ National Nanjing New Drug Screening Center, China Pharmaceutical University, Nanjing, China; ^3^ Drug Discovery and Design Center, State Key Laboratory of Drug Research, Shanghai Institute of Materia Medica, Chinese Academy of Sciences, Shanghai, China

**Keywords:** acetylation, camptothecin, SIRT1, superoxide dismutase, oxidative stress

## Abstract

Cancer cells are characterized by a high dependency on antioxidant enzymes to cope with the elevated rates of reactive oxygen species (ROS). Impairing antioxidant capacity in cancer cells disturbs the ROS homeostasis and exposes cancer cells to massive oxidative stress. In this study, we have discovered that superoxide dismutase 1 (SOD1), a major player in maintaining the cellular redox status, was acetylated at lysine 71. This acetylation, which was primarily deacetylated by Sirtuin 1 (SIRT1), suppressed the enzymatic activity of SOD1 via disrupting its association with copper chaperone for SOD1 (CCS). More importantly, genotoxic agents, such as camptothecin (CPT), induced SOD1 acetylation by disrupting its binding with SIRT1. CPT-induced SOD1 acetylation was stimulated by its provoked ROS, suggesting a positive feedback loop, in which ROS per se impairs the antioxidative defence of cancer cells and reinforces oxidative stress stimulated by anticancer agents. The intrinsic abundance of SOD1 acetylation varied among cancer cells, and high level of SOD1 acetylation was correlated with elevated sensitivity to CPT. Together, our findings gained mechanistic insights into how cytotoxic agents fine tune the intracellular ROS homeostasis to strengthen their anticancer effects, and suggested SOD1 acetylation as a candidate biomarker for predicting response to CPT-based chemotherapy.

## INTRODUCTION

Regulation of reactive oxygen species (ROS) homeostasis plays key roles in living organisms [1, 2]. A moderate increase in ROS promote cell proliferation and differentiation, while the excessive amounts of ROS causes oxidative damage to lipids, proteins and DNA and induces cells undergoing apoptosis [3]. Cancer cells are characterized by persistently higher levels of ROS than non-transformed cells due to increased metabolic activity and the dysregulation of redox balance, which renders cancer cells more vulnerable to oxidative stress and a high dependency on antioxidant enzymes to detoxify from ROS [4]. As such, modulation of ROS homeostasis, via an increase in ROS levels or impairing antioxidant capacity, has been considered as an important strategy for cancer therapy [5–7].

Superoxide dismutase 1 (SOD1), which is involved in the conversion of toxic superoxide anions into molecular oxygen and hydrogen peroxide, is an important member in the intracellular ROS-scavenging system [8]. Active, mature SOD1 is a homodimeric protein containing two zinc (Zn^2+^) and two copper (Cu^2+^) ions for its stability and activity. The association with the copper chaperone for SOD (CCS) is essential for the activation of copper/zinc SOD, although an additional minor CCS-independent pathway has been reported in mammals [8]. CCS specifically delivers Cu to SOD1, which allows the formation of an intrasubunit disulfide bond between SOD1^Cys-57^ and SOD1^Cys-146^, and results in an enzymatically active homodimers of SOD [9, 10]. Thus far, CCS binding remains the most dominant mechanism for the regulation of the enzymatic activity of SOD1. Apart from CCS association, increasing evidence has indicated that diverse post-translational modifications, including nitration [11], phosphorylation [12], glutathionylaion [13] and glycation [14], are involved in the regulation of the dismutase activity of SOD1. Post-translational modifications have emerged as an important aspect in fine-tuning the signal process of SOD1 involved redox homeostasis. In the meanwhile, we have noticed that recent global proteomic profiling has identified lysine acetylation as a frequently occurred modification for cytoplasmic proteins, in particular metabolic enzymes including SOD1 [15–17], but the cellular functions of these modifications are still unknown.

This study started from the validation of occurrence of SOD1 acetylation in cancer cells, and focused on the investigation of the biological significance of SOD1 acetylation. Our findings provided first evidence revealing the role of acetylation in modulating the SOD1 activity. The study highlighted a SOD acetylation mediated positive feedback loop in strengthening oxidative stress caused by genotoxic anticancer agents, and suggested the translational value of SOD1 acetylation for camptothecin-based chemotherapy.

## RESULTS

### SOD1 is acetylated at lysine 71

**Figure 1:**
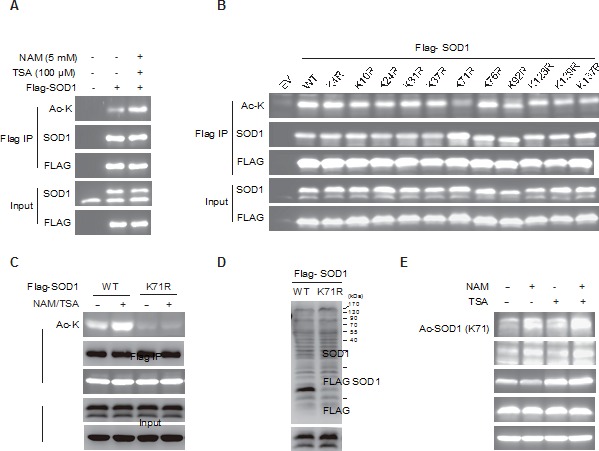
SOD1 is acetylated on K71 residue **A.** Acetylation of exogenous SOD1. Ectopically expressed SOD1 was immunopreciptated and acetylation of SOD1 was examined using a pan-acetyl-lysine antibody. Cells were pretreated with TSA (500 nM) or/and NAM (10 mM) for 12 hr prior to immunoprecipitation; **B.**, **C.** Impact of lysine mutant on SOD1 acetylation. **B.**, wildtype (WT) SOD1 or indicated lysine mutants was transfected into cells and acetylation of each mutant was examined as described in **A.**. EV, empty vector; **C.**, Acetylation of ectopically expressed SOD1WT, SOD1K71R, SOD1K71Q with or without TSA (500 nM) and NAM (10 mM) treatment was analyzed; **D.** Specificity of anti-acetyl-K71 antibody. Flag-tagged SOD1 WT or K71R mutant was transfected into HCT116 cells and acetylation of each purified protein was measured by immunoblotting using an anti-Ac SOD1 (K71) antibody; **E.** Endogenous SOD1 is acetylated at K71. Cells treated with NAM and TSA was analyzed by anti-Ac SOD1 (K71) antibody.

A number of mass spectrometry-based proteomic studies have suggested the occurrence of acetylation on SOD1 [15–17], but there lacks evidence to support acetylation of endogenous SOD1, and the biological significance of this modification remains unclear. We firstly validated the acetylation of SOD1 using a pan-specific anti-acetylated lysine antibody in cancer cells with ectopically expressed SOD1. Acetylation was detected on flag-tagged SOD1 enriched from HCT116 colon cancer cells. Treatment of protein deacetylase inhibitors, namely nicotinamide (NAM) and Trichostatin A (TSA), resulted in an increase in the acetylation of SOD1 (Figure 1A).

We next determined the main lysine sites where the acetylation occurred. SOD1 contains 11 lysine (K) residues, which are K4, K10, K24, K31, K37, K71, K76, K92, K123, K129 and K137. As lysine lysine (K)-arginine (R) replacement is widely used to generate acetylation-deﬁcient mutants [18–20], each of the lysine was individually mutated to a nonacetylatable arginine, and the impact on SOD1 acetylation was examined. Among the 11 mutants, only the K71R mutation largely abolished SOD1 acetylation (Figure 1B) and the treatment of deacetylase inhibitors failed to increase the detectable signaling of acetylation (Figure 1C), indicating the acetylation of SOD1 occurred at K71. Meanwhile, alignment of SOD1 protein sequence revealed that K71 was evolutionarily conserved across diverse species (Supplemental Figure S1). Further, we generated an antibody that specifically recognized SOD1 bearing acetylation at K71. The antibody was able to detect the acetylation of ectopically expressed wild-type SOD1 but not the K71R mutant (Figure 1D), demonstrating the selectivity of the antibody. This antibody enabled the first detection of the acetylation of endogenous SOD1, which was significantly increased by treatment of deacetylase inhibitors NAM plus TSA (Figure 1E). Meanwhile, SOD1 depletion by two independent siRNAs significantly decreased the SOD1 acetylation enriched by deacetylase inhibitors, further supporting the specificity of this antibody (Supplemental Figure S2). These results together demonstrated the acetylation of SOD1 at K71, which intrigued us to explore the biological significance of SOD1 acetylation.

### Acetylation inactivates the dismutase activity of SOD1

**Figure 2:**
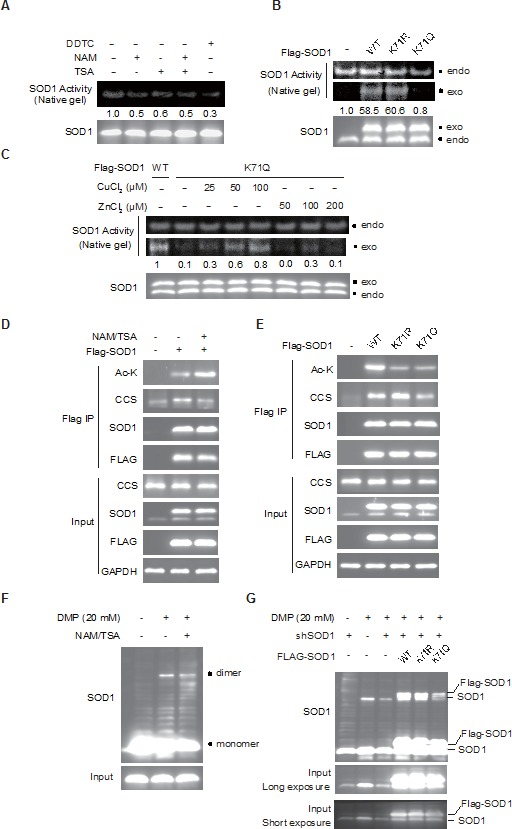
SOD1 acetylation inactivates SOD1 **A.** SOD1 activity is decreased by NAM/TSA treatment. Cells were treated by TSA (500 nM) and NAM (10 mM) for 12 hr and SOD1 activity was determined using an in-gel SOD1 activity assay; **B.** Mutations of the acetylation lysine residues affect SOD1 activity. Flag-tagged SOD1 WT, K71R or K71Q mutant was transfected into HCT116 cells and SOD1 activity was measured as in **A.**. endo, endogenous SOD1, exo, Flag-tagged SOD1; **C.** Sufficient supply of copper rescues the activity of K711Q mutant; **D.** The interaction between SOD1 and CCS is decreased by NAM/TSA treatment. HCT-116 cells transfected with Flag-tagged SOD1 were treated with NAM/TSA for 12 hr. The presence of CCS in the immunoprecipitated protein complexes was assessed by immunoblotting; **E.** Acetylation mimetic mutation at K71 decreased the interaction between SOD1 and CCS; **F.** The level of SOD1 homodimers is reduced under NAM/TSA treatment. HCT116 cells were treated with NAM/TSA for 12 hr. Cells lysis in the presence or absence of DMP (20 mM) were assessed by immunoblotting using SOD1 antibody; **G.** Acetylation mimetic mutation at K71 decreased the level of SOD1 homodimers. HCT116 cells stably knock-down SOD1 (shSOD1) were transfected with Flag-tagged SOD1 WT, SOD1K71R or SOD1K71Q. SOD1 homodimers were detected as described in **C.**.

We asked whether acetylation of SOD1 affected its enzymatic activity. The dismutase enzymatic activity of SOD1 was measured using a specific in-gel enzymatic activity assay using the native polyacrylamide gel electrophoresis. Treatment with deacetylase inhibitors NAM or TSA, similar to SOD1 inhibitor DDTC, resulted in the reduction of SOD1 activity while the SOD1 protein level was not affected in parallel (Figure 2A), suggesting that acetylation of SOD1 negatively regulates the SOD1 activity. For further confirmation, we compared the enzymatic activity of wild type SOD1, K71R mutant and acetylation mimetic K71Q mutant. Flag-tagged wild type or mutant constructs was transfected into HCT-116 cells, and the enzymatic activity of endogenous and exogenous SOD1 was differentiated by their diverse migration in the native polyacrylamide gel electrophoresis. K71R mutant behaved similar to wildtype SOD1 in the activity assay, whereas the K71Q mutant showed a significant decrease in the catalytic activity (Figure 2B). These results suggested acetylated SOD1 as an inactive form of SOD1.

### Acetylation of SOD1 disrupts its interaction with CCS

We then asked how acetylation affected the SOD1 activity. To address this question, we inspected the multi-step process of SOD1 maturation, which involves zinc binding, copper loading by CCS, and homodimerization prior to turning into an active homodimeric enzyme. We firstly examined whether the impaired SOD1 activity was due to the impaired zinc or/and copper loading, which initiates the process of SOD1 maturation. To this end, the acetylation mimetic K71Q mutant was incubated with increasing amount of zinc or copper to examine whether the deficient SOD1 activity could be rescued by sufficient zinc/copper supplies. Indeed, we observed that copper incubation instead of zinc incubation was able to reverse the enzymatic activity of K71Q mutant to the similar level of wildtype SOD1 (Figure 2C). This data largely excluded the possibility of impaired zinc loading of the K71Q mutant, and led us to speculate that acetylation of SOD1 probably affected its interaction with CCS, a SOD1 binding partner specifically responsible for copper delivery. As such, flag-tagged SOD1 was transfected into HCT-116 cells and the interaction between SOD1 and CCS were assessed using co-immunoprecipitation assay. It was found that treatment with NAM and TSA, which effectively enriched cellular SOD1 acetylation, largely disrupted the interaction between SOD1 and CCS (Figure 2D). Further, we compared CCS binding ability between SOD1 wildtype and the two acetylation relevant mutants. The interaction between CCS and the acetylation defective K71R mutant was enhanced compared with that of wildtype SOD1, whereas K71Q mutant exhibited decreased association with CCS (Figure 2E).

CCS binding and it mediated copper loading are required for the subsequent homodimerization and ultimate activation of SOD1. Therefore, we also examined the impact of acetylation on SOD1 homodimerization. The dimerized SOD1 was visualized by the treatment with cross-linking reagent dimethyl pimelimidate (DMP) prior to denatured polyacrylamide gel electrophoresis. SOD1 dimmers were recognized as a subset with shifted molecular weight. In agreement with our results above, deacetylase inhibition using NAM and TSA decreased the proportion of endogenous SOD1 homodimers (Figure 2F). Further, SOD1 wildtype, K71R or K71Q mutant was transfected into HCT-116 cells, where endogenous SOD1 was depleted using short hairpin (shRNA) to eliminate the interference of endogenous band for recognizing ectopically expressed proteins. Compared with the wildtype SOD1, acetylation mimetic mutant SOD1 K71Q showed a considerable decrease in the proportion of homodimers, whereas K71R barely effected SOD1 dimmers formation (Figure 2G).

Together, we concluded that SOD1 acetylation at K71 disrupted the interaction between SOD1 and CCS, which impaired formation of SOD1 homodimers, and in turn attenuated the enzymatic activity of SOD1.

### SIRT1 deacetylates SOD1 acetylation

**Figure 3:**
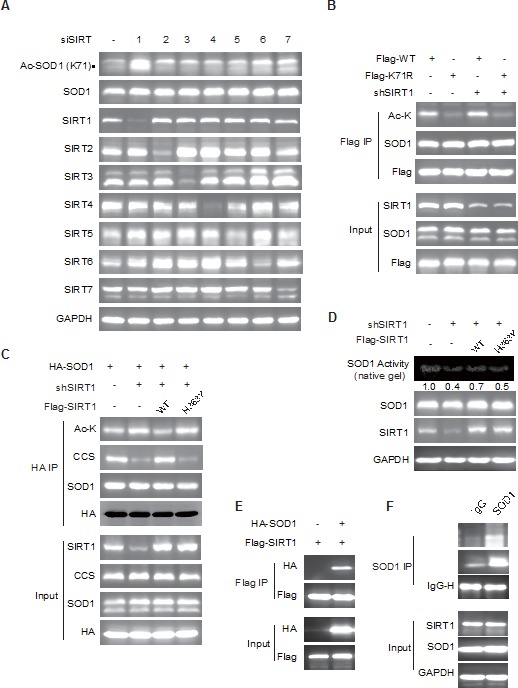
SIRT1 deacetylates SOD1 **A.** SIRT1 knockdown increased SOD1 acetylation. Cells were transfected with siRNA against SIRT1 to 7 and SOD1 acetylation was detected by anti-Ac SOD1 (K71) antibody; The Ac-SOD1 band was indicated by the solid triangle. **B.** SIRT1 deacetylated SOD1 acetylation on K71. HCT116 ShSIRT1 cells were transfected with flag-tagged SOD1 wildtype (WT) or K71R mutant. SOD1 acetylation was detected by using a pan-acetyl-lysine antibody following anti-Flag immunoprecipitation; **C.** Catalytic activity of SIRT1 is required for the deacetylation of SOD1. HA-tagged SOD1 was co-transfected with Flag-tagged SIRT1 wildtype (WT) or catalytically inactive mutant H363Y into SIRT1-depleted HCT-116 cells. Acetylation was detected as in **B.**, **D.** Catalytic activity of SIRT1 is required for the regulation of SOD1 activity. Endogenous SOD1 activity was measured in shSIRT1 cells reconstituted with SIRT1 WT or H363Y mutant; **E.**, **F.** SOD1 interacts with SIRT1. E, HA-tagged SOD1 was co-transfected with Flag-tagged SIRT1 into HCT-116 cells. Co-immunoprecipitation was performed using anti-Flag agarose. The presence of SOD1 in the immunoprecipitate was assessed by immunoblotting. F, interaction of endogenous SOD1 and SIRT1. Endogenous SOD1 was immunoprecipitated with a rabbit polyclonal antibody against SOD1. Rabbit Immunoglobulin G (IgG) was used as a negative control. The presence of SIRT1 in the immunoprecipitate was assessed by immunoblotting with an antibody against SIRT1.

Protein acetylation is critically regulated by deacetylases, which are often dysregulated in cancer cells and result in aberrant acetylation status in cancer cells [21, 22]. To identify responsible deacetylases may help understand the physiological significance of SOD1 acetylation. HDACs and sirtuins represent the two major classes of protein deacetylases and can be respectively inhibited by the pan-inhibitor TSA and NAM. According to our results in Figure 1E, NAM treatment considerably increased the level of SOD1 K71 acetylation whereas TSA treatment only had a marginal effect, suggesting that sirtuin family members are primarily involved in SOD1 deacetylation. To further identify the involved sirtuin(s), endogenous sirtuin 1 (SIRT1) to 7 was individually depleted using specific siRNAs followed by the detection of SOD1 acetylation. Among the 7 sirtuins, only SIRT1 depletion dramatically increased endogenous level of SOD1 acetylation at K71 (Figure 3A and Supplemental Figure S3). SIRT1 depletion enriched the acetylation of the wildtype SOD1 but not that of the K71R mutant (Figure 3B), suggesting a dominant role of SIRT1 in the regulation of SOD1 acetylation at K71. Further, the enhanced SOD1 acetylation by SIRT1 knockdown was reversed by the ectopic expression of wildtype SIRT1 but not the catalytically inactive H363Y mutant. The alteration in the SOD1 acetylation was associated with the interaction change between SOD1 and CCS (Figure 3C). The impact of SIRT1 on SOD1 acetylation was also reflected by the enzymatic activity of SOD1. Reconstitution of wildtype SIRT1 in SIRT1 stably depleted cells partially rescued the suppressed SOD1 activity by SIRT1 downregulation, but the effect of H363Y mutant was indiscernible (Figure 3D). In support of all these observations, we detected the interaction between SOD1 and SIRT1, as shown by the detection of SIRT1 in the immunocomplex pull-downed by Flag-tagged SOD in HCT-116 cells (Figure 3E). We also tested whether the SOD1 and SIRT1 was able to interact endogenously. Immunoprecipitation of endogenous SOD1 using an anti-SOD1 antibody revealed the interaction of SOD1 with endogenous SIRT1 in HCT-116 cells (Figure 3F).

These findings collectively indicated that SIRT1 deacetylates SOD1 at K71, which promotes its interaction with CCS, and enhances the enzymatic activity of SOD1.

### Genotoxic agents induce SOD1 acetylation via ROS generation

**Figure 4:**
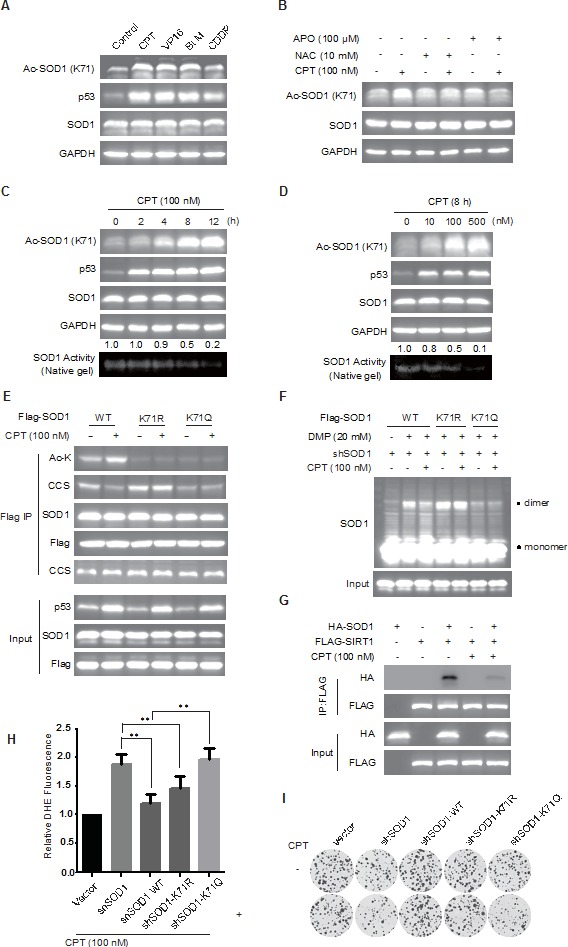
Genotoxic agents promote SOD1 acetylation and inactivate SOD1 **A.**, Genotoxic agent induces SOD1 acetylation. HCT-116 were treated with camptothecin (CPT, 100 nM), etoposide(VP-16, 10 μM), bleomycin (BLM, 10 μM) or cisplatin (CDDP, 20 μM) for 12 hr and SOD1 acetylation was examined using an anti-Ac SOD1 (K71) antibody; **B.** CPT-induced SOD1 acetylation is mediated by ROS. Cells were pretreated with or without NAC or APO for 2 hr before exposure to CPT (100 nM, 12 hr); **C.**, **D.** The impact of CPT treatment on SOD1 acetylation and enzymatic activity. Time-dependent **C.** and dose-dependent **D.** induction of SOD1 acetylation and corresponding alteration in SOD1 activity was measured using immunoblotting or an in-gel enzymatic assay; **E.** The impact of CPT treatment on the interaction between SOD1 and CCS. Flag-tagged SOD1 WT, K71R, K71Q was transfected into HCT116 cells treated with or without 100 nM CPT for 12 hr. The presence of CCS in the immunoprecipitated protein complexes was assessed by immunoblotting. **F.** The impact of CPT on the formation of SOD1 homodimers. Flag-tagged SOD1 WT, K71R, K71Q was transfected into HCT116 cells treated with or without 100 nM CPT for 12 hr; **G.** The impact of CPT on the interaction between SOD1 and SIRT1. HA-tagged SOD1 were co-transfected with Flag-tagged SIRT1 into HCT-116 cells; **H.** The impact of SOD1 acetylation on cytosolic superoxide levels under CPT stimulation. HCT116 cells stably knock-down SOD1 (shSOD1) were transfected with Flag-tagged SOD1 (WT), SOD1K71R or SOD1K71Q, and cells were treated with CPT. The cytosolic superoxide level was measured by DHE staining and FACS analysis. Data represent mean ± SEM (*n* = 3), **P < 0.01. **I.** SOD1 Acetylation promotes cells sensitive to CPT treatment. The sensitivity of cells HCT116 shSOD1 cells expressing the wildtype, K71R or K71Q SOD1 were treated with CPT (1 nM) for 14 days and measured by a cologeneic assay.

SIRT1 is essentially involved in coping with various stress including oxidative stress, while SOD1 plays a key role in scavenging cellular ROS [23], a natural byproduct of the normal oxygen metabolism but dramatically increased in environmental stress such as chemotherapy. Indeed, mounting evidence has suggested that cytotoxic anticancer agents induced oxidative stress contribute to the anticancer efficacy of these agents [24–29]. These information together implicate a possible involvement of SOD1 acetylation in cytotoxic agents caused oxidative stress. We then treated HCT-116 cells with various genotoxic anticancer agents including cisplatin (CDDP), camptothecin (CPT), etoposide (VP16) and bleomycin (BLM) to test the possible impact on SOD1 acetylation. Treatment with these agents all considerably increased SOD1 acetylation (Figure 4A). Interestingly, the impact on the increase of SOD1 acetylation was correlated with the level of ROS accumulation caused by these agents (Supplemental Figure S4A and S4B). Moreover, pretreatment with ROS scavenger N-acetylcysteine (NAC) or inhibition of NADPH oxidase using apocynin (APO), apparently reversed DNA damaging agents induced SOD1 acetylation (Figure 4B), implicating that genotoxic stress associated ROS generation accounted for the enhanced SOD1 acetylation.

We then used CPT as a representative to follow up the impact of DNA damage on SOD1 acetylation. CPT caused DNA damage, as reflected by p53 upregulation, induced SOD1 acetylation in a dose- and time-dependent manner. The induced increase of SOD1 acetylation was closely associated with a decline of SOD1 activity (Figure 4C and 4D), and the interaction with CCS (Supplemental Figure S5A and S5B). Consistently, CPT treatment disrupted the interaction between SOD1 and CCS but failed to affect the CCS binding to K71R mutant, suggesting an acetylation-dependent impact on CCS binding (Figure 4E). In line with these findings, CPT treatment suppressed dimerization of wildtype SOD1 but did not affect either K71R or K71Q mutant (Figure 4F, Supplemental Figure S6A and S6AB). Importantly, we also observed that treatment with CPT largely disrupted the interaction between SOD1 and SIRT1 (Figure 4G), which may explain the enhanced SOD1 acetylation upon DNA damage. Together, these data provided the first evidence showing that chemotherapies induce SOD1 acetylation and impair its enzymatic activity in cancer cells, which may result from disrupted interaction with SIRT1.

SOD1 is essential for maintaining the redox homeostasis of cancer cells. CPT treatment increased ROS generation in cancer cells (Supplemental Figure S4B) whilst suppressed SOD1 activity (Figure 4C and 4D). This may suggest the further enhanced oxidative stress caused by SOD1 inactivation. We hence examined the impact of acetylation of SOD1 on cytosolic ROS level in HCT-116 cells, where the endogenous SOD1 was depleted using shRNA. shRNA-resistant SOD1 wild-type, K71R and K71Q SOD1 were transfected into HCT-116 cells respectively followed by the treatment of CPT. As expected, SOD1 depletion led to a dramatic increase of intracellular ROS level. Ectopic expression of wildtype SOD1 was able to rescue the ROS generation resulted from SOD1 depletion. In contrast, the acetylation-mimetic K71Q mutant, with impaired enzymatic activity, failed to reverse the intracellular ROS level (Figure 4H). However, it appeared puzzling that the nonacetylatable K71R mutant behaved similar to the wildtype SOD1 in ROS clearance upon CPT treatment. We speculated that this was likely due to the small proportion of acetylated form induced by CPT among total amount of exogenous SOD1. Indeed, cell sensitivity to CPT treatment measured by a cologeneic assay was consistent with results of the ROS clearance. Cells expressing acetylation-mimetic SOD1 K71Q mutant were more sensitive than either wild-type SOD1 and K71R mutant to CPT treatment (Figure 4I).

### SOD1 acetylation sensitizes cancer cells to DNA damaging agents

**Figure 5:**
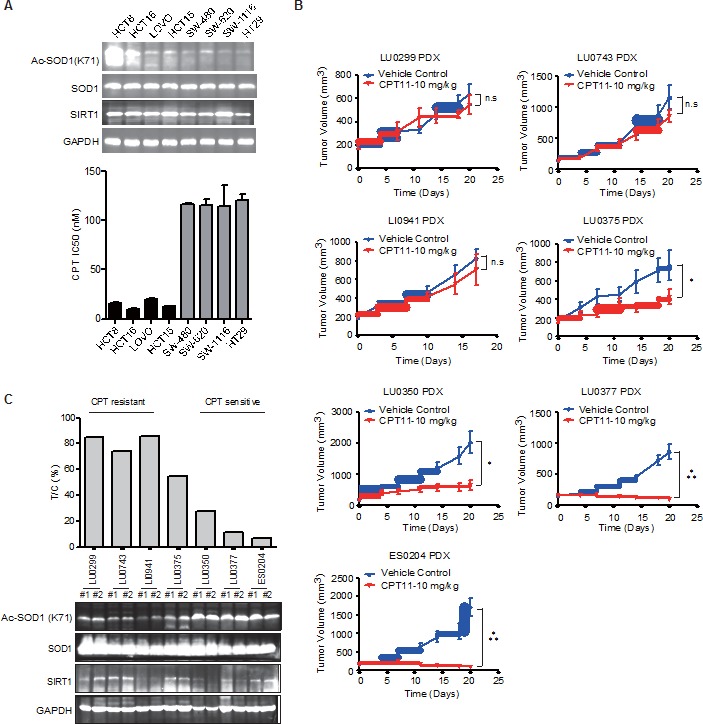
SOD1 acetylation is associated with the response to CPT treatment **A.** SOD1 acetylation and cell sensitivity to CPT-11 in colon cancer cell lines. **B.**, **C.** Intratumoral SOD1 acetylation level and response to CPT-11 treatment of patient-derived xenograft models. **B.** Growth curves of tumors from indicated PDX models treated with vehicle control or 10 mg/kg CPT-11 daily for 21 consecutive days. Data represent mean tumor volume ± SEM (*n* = 6), ***P < 0.001; *P < 0.05; n.s., not significant. **C.** Correlation between SOD1 K71 acetylation and sensitivity to CPT-11. T/C%, relative tumor volume versus vehicle control on day 21.

The substantial impact of K71Q mutant shown above suggests a possibility that the abundance of SOD1 acetylation may be a determinant of the sensitivity to the CPT-based chemotherapies, which are used in the clinical therapy of various types of human cancers including the first line treatment for colon cancer. We then probed the status of SOD1 acetylation at K71 in a panel of colon cancer cells, and found that the basal level of SOD1 acetylation varied largely across the cells (Figure 5A). Some cells lines, like HCT-8 and HCT-16, displayed massive abundance of intrinsic SOD1 acetylation. These data suggest that SOD1 acetylation status may confer a distinct antioxidant capacity across cancer cells, and those with low capacity may be more susceptible to CPT-induced oxidant stress. Indeed, we found that cells with high SOD1 acetylation were relatively more sensitive to CPT treatment. We also tested whether Ac-SOD1 level alteration in responses to CPT was correlated with the CPT sensitivity of these cells as well. Ac-SOD1 level was examined after 12 hr exposure to CPT treatment in the colon cancer cell lines (Supplemental Figure S7). It was found that cell with higher basal level of Ac-SOD1 showed more significant increase of Ac-SOD1 level upon CPT treatment, suggesting a correlation between Ac-SOD1 level change and the response to CPT treatment.

Apart from colon cancer, we also tested whether basal Ac-SOD1 levels were correlated with the sensitivity to CPT treatment in lung cancer cells. The sensitivity of 13 lung cancer cells towards topotecan, a CPT analogue, was extracted from Cancer Cell Line Encyclopedia (CCLE) database. Immunoblotting detection of Ac-SOD1 level from those cells revealed that the basal level of Ac-SOD1 was correlated with the sensitivity to CPT treatment in lung cancer cells (Supplemental Figure S8). This data suggested that correlation of Ac-SOD1 and camptothecin-sensitivity could be a general mechanism beyond colon caner

For further confirmation, we proceeded to validate this finding in patient-derived xenograft (PDX) models, which are believed to faithfully resemble the characteristics of human tumors in many aspects including heterogeneity, histology and genetic alterations [30–33]. CPT-11 efficacy was screened in a small panel of PDX models across different cancer types including lung cancer (LU0299, LU0743, LU0375, LU0350, LU0377), liver cancer (LI0941) and esophageal cancer (ES0204). We observed the diverse response of these models to CPT-11 treatment and subgrouped the models into CPT sensitive and resistant subset (Figure 5B). Meanwhile, we also measured the basal level of SOD1 acetylation on K71. The models with higher level of SOD1 acetylation were more responsive to the treatment (Figure 5C). These results suggest a potential value of SOD1 K71 acetylation in stratifying the responsive subset to CPT-11 based chemotherapy.

## DISCUSSION

**Figure 6:**
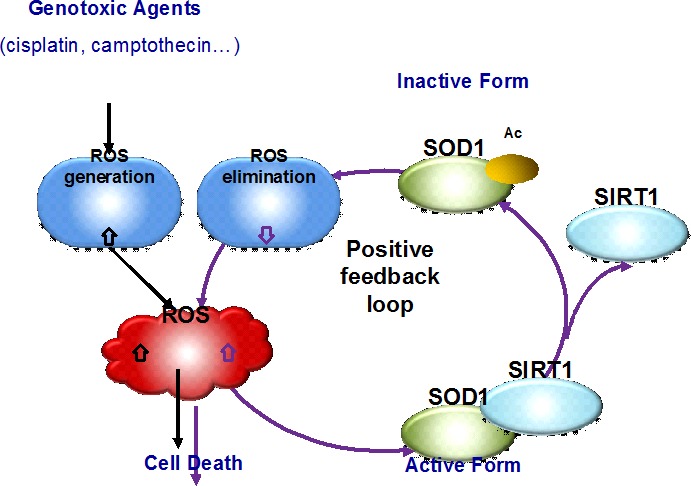
A schematic model showing the positive feedback of ROS induction resulted from SOD1 acetylation

The increased generation of ROS and altered redox status in cancer cells offers an interesting therapeutic window that cancer cells are more sensitive than normal cells to agents causing further accumulation of ROS [4]. In fact, direct or indirect affects on ROS amount have been widely believed to contribute to the anticancer efficacy of cytotoxic anticancer agents, in particular genotoxic agents. Generation of high levels of ROS has been observed in patients receiving various chemotherapy treatment [24–29], though the mechanism of ROS generation may vary among the agents [34]. Apart from the widely studied ROS generation, the molecular insights into the ROS homeostasis changes by genotoxic agents have been very limited. In this study, we have provided the first evidence showing that genotoxic agents caused ROS accumulation was able to impair the antioxidant capacity of cancer cells via diminishing the activity of antioxidant enzyme SOD1. Our findings suggest the existence of a positive feedback mechanism in which ROS per se mediates the impairment of the antioxidative enzyme (defence) system of cancer cells (Figure 6). The feedback inhibition of SOD1 further raises the cytosolic ROS level, reinforces oxidative stress, and promotes the effectiveness of the anticancer agents.

It has long been noticed that the increase of ROS level and DNA damage, can be found one being caused by the other one; ROS induces DNA damage while DNA damage agents could also increase ROS generation. Cytotoxic anticancer agents, including cisplatin, mitomycin C, doxorubicin, CPT and ultraviolet radiation induced ROS are important for the induction of cell apoptosis and anticancer efficacy of these agents [24–29]. While in certain cancer cells, chemotherapeutic agents induced persistent ROS stress may induce adaptive stress responses including activation of redox-sensitive transcription factors, leading to an increase in the expression of ROS-scavenging enzymes, such as SOD and glutathione, to counteract with ROS stress. All these events enable cells to survive with the high level of ROS and render cancer cells more resistant to chemotherapeutic agents [6, 35]. Accordingly, modulating ROS-scavenging enzymes activity could enhance the anti-tumor activity of genotoxic agents via ROS mediated apoptosis induction. Intriguingly, our findings provided new insights by showing an apposing mechanism, in which the genotoxic agents, in parallel to ROS induction, are able to paralyze the antioxidant defence of cancer cells to facilitate their anticancer efficacy. Our findings are particularly interesting given the fact that cancer cells often maintain a high antioxidant capacity to cope with the massive ROS resulted from fast growth. This finding highlighted the role of antioxidant defence system in determining the efficacy the genotoxic anticancer agents, and may lead to a better understanding of the anticancer mechanism of genotoxic agents.

The key molecular mechanism behind involves the acetylation of SOD1 on the lysine 71 residue. We have shown that acetylation decreases SOD1 activity by impairing the interaction between SOD1 and CCS, and hence decreasing the output of enzymatically active SOD1 homodimers in the maturation process of SOD1. We also noticed that the mutation of lysine 71 to arginine, which abolished the acetylation of SOD1, did not obviously promote the activity of SOD1. One possible explanation is that most cancer cells feature a high antioxidant capacity, in which SOD1, as a critical anti-oxidant enzyme, may maintain a high basal activity and can hardly to be further enhanced. Likewise, we have observed that the acetylation mimetic K71Q mutant exhibits striking impact on CPT-induced ROS generation and cell survival whereas the K71R mutant did not obviously promote scavenging of ROS or cell viability. We speculated that this might also partially result from the small proportion of acetylated form induced by CPT among total amount of exogenous SOD1.

We also exploited how SOD1 K71 acetylation decreases CCS interaction with SOD1. As the human SOD1-CCS complex structure has not been solved yet, we took advantage of the solved crystal structure of yeast SOD1-CCS complex (ySOD1-yCCS) (PDBID: 1JK9) [36] and aligned the human SOD1 to yeast SOD1 in the ySOD1-yCCS crystal structure (PDBID:1JK9) using PyMol software. According to the structural alignment (Supplemental Figure S9), hSOD1 shares high structural similarity with ySOD1, and K71 (T71 in yeast SOD1) does not lie in the protein-protein interaction surface. Also, a previous crystallography study has suggested that the binding between SOD1 and CCS may involve conformational changes. We speculated that K71 acetylation may interfere with this conformational change through allosteric regulation and hinder its binding to CCS1.

Previous studies have suggested two possible mechanisms by which CPT treatment may influence SOD1 acetylation. Genotoxic stress was reported to inactive SIRT1 via the ATM/ATR-DBC1 signaling [37, 38]. To test this possibility, we examined the SOD1 acetylation in ATM knockdown cells but did not observe the apparent reduction in either DBC1 phosphorylation or SOD1 acetylation, which largely ruled out the possible involvement of this pathway (Supplemental Figure S10). Alternatively, ROS induced by CPT is known to inhibit SIRT1 activity by evoking oxidative modifications on its cysteine residues [39]. In this case, we did not observe the alteration of SIRT1 activity upon CPT treatment (Supplemental Figure S11). Instead, our data suggested that CPT disrupted the interaction between SIRT1 and SOD1, which may possibly result from oxidative modification of SIRT1 by ROS.

SIRT1 is known to play a critical role in coordinating the cellular response to stress. SIRT1 expression and activity are regulated by cellular stressors, including metabolic, genotoxic, oxidative and phototoxic stress. SIRT1 impacts cell survival by deacetylating substrate proteins to drive the cell towards a cytoprotective pathway [40]. Moderate overexpression of SIRT1 provides cells protection against oxidative stress by increasing the activity of catalase [41]. SIRT1 may also protect against oxidative stress through the modulation of family of forkhead transcription factors (FOXO) [42, 43] and the induction of manganese SOD (MnSOD) expression [44]. Our result supported and complemented the previous studies by showing that SIRT1 also regulates the oxidative metabolism via directly deacetylating and modulating the activity of SOD1. All these evidence suggests an important role of SIRT1 in determining the antioxidant capacity of cancer cells and hence the outcome of chemotherapy. Indeed, a previous study has observed the correlation between SIRT1 expression level and the CPT sensitivity in prostate cancer cells, although in very limited number of cell lines [45]. An apparent caveat is that SIRT1 expression level may not be necessarily associated with its activity. Indeed, we have observed that in colon cancer cell lines and the PDX tumors, SIRT1 protein level was not correlated with the CPT sensitivity (Figure 5A and 5C). According to our results, the basal level of SOD1 acetylation varies largely among either the cancer cell lines or patients tumor tissues; high level SOD1 acetylation is closely correlated with the increased response to CPT treatment. We speculate that while cancer cells often feature an increased antioxidant capacity, high level of SOD1 acetylation represents an intrinsic silencing of SOD1, and is also an indicator of low activity of SIRT1. Thus abundant basal level of SOD1 acetylation is able to stratify the subset with low capacity to copy with oxidative stress of cancer cells. The clinical value of SOD1 acetylation may deserve further investigation in clinical practice to increase the response rate of CPT-based chemotherapy regimen.

## MATERIALS AND METHODS

### Plasmids

The FLAG/HA-tagged form of SOD1 was generated by subcloning Xho I-Hind III an cassette of SOD1 into the Flag/HA-pcDNA3.1 mammalian expression vector. The plasmid pECE encoding SIRT1/SIRT1-H363Y with a FLAG tag was purchased from Addgene. The RNAi Consortium (TRC) Lentiviral shRNAs against SOD1 (Clone ID: TRCN0000039808, targeting the 3′UTR region of SOD1) and against SIRT1 (Clone ID: TRCN0000039808) were purchased from Thermo. Mutations in pcDNA3.1-SOD1-FLAG were introduced by the change site directed mutagenesis kit (Saibaisheng Gene Technolog, Shanghai, China). Sequences were verified by automated sequence analysis (Sangon Biotech, Shanghai, China).

### siRNA transfection

For siRNA transfection, HCT116 cells were plated at 3×10^5^ cells/ml in OPTI-MEM serum-free medium and transfected with siRNA duplex using Lipofectamine RNAiMAX Reagent Agent (Life Technologies) according to the manufacturer's instructions. siRNAs were ordered from Sigma-Aldrich. The sequences were as follows: siSOD1 #1: TTC GAG CAG AAG GAA AGT AAT GGA CCA dTdT; siSOD1 #2: GGC CUG CAU GGA UUC CAU G dTdT.

### Cell culture

Human colon cancer HCT-116 cells purchased from American Type Culture Collection (ATCC) were cultured in McCOY's 5A medium (Life Technology) supplemented with 10% FBS. HCT116 cell lines stably transfected with short hairpin RNA targeting SOD1 or SIRT1 (ThermoFisher) (shSOD1/shSIRT1) were constructed according to manufacturer's instructions and maintained in McCOY's 5A medium supplemented with 1 μg/μl puromycin dihydrochloride (Sigma).

### Immunoprecipitation assay

Immunoprecipitation of Flag-tagged SOD1 was carried out using anti-FLAG M2 beads. Equal amounts of proteins in lysis Buffer were used for precipitation. Input samples represent ~1% of protein amounts used for immunoprecipitation. The following antibodies were used for immunoprecipitation and followup immunoblotting: monoclonal rabbit anti-SOD1 (Epitomics); monoclonal rabbit anti-Sir2/SIRT1 (Epitomics); monoclonal mouse anti-acetylated-Lysine(Cell Signaling Technology); monoclonal mouse anti-P53 (Santa Cruz Biotechnology); monoclonal mouse anti-CCS (Santa Cruz Biotechnology); polyclonal mouse anti-FLAG M2 affinity Gel (Sigma); monoclonal mouse anti-DYKDDDDK-Tag (Abmart, Shanghai, China); monoclonal mouse anti-HA-tag (Abmart); monoclonal rabbit anti-GAPDH (Epitomics). Antibodies specifically recognizing acetylation at lysine 71 were prepared by PTM BioLab, Inc. (Hangzhou, China).

### In-gel SOD1 activity assay

Cells were harvested and lysed in lysis buffer (25 mM HEPES; pH 7.9, 100 mM NaCl, 1 mM EDTA, 1% Triton X-100, 10% Glycerol, 1 mM Na_3_VO_4_, 1 mM PMSF and protease inhibitors). The supernatant was subjected to non-denaturing gel electrophoresis and nitro blue tetrazolium (NBT, Beyotime Institute of Biotechnology, Nanjing, China) staining to examine SOD1 activity as described previously with slight modifications [9]. Cell lysate was loaded directly without boiling to a nondenaturing 12% polyacrylamide gel. After electrophoresis, the gel was incubated for 45 min in the dark in 50 mM potassium phosphate buffer (pH 7.8) supplemented with 0.34 mM NBT and 14 mM riboflavin (Beyotime Institute of Biotechnology). The gel was subsequently exposed to light for 15 min after 1 μl/ml TEMED was added to the reaction mixture.

### Assessment of intracellular superoxide anions

Cells were incubated with 3 μM dihydroethidium (hydroethidine, DHE; Invitrogen) in PBS for 30 min at 37°C following the treatment with CPT at 100 nM for 12 hr. Cells were washed once with PBS, trypsinized, and immediately analyzed using flow cytometry on a FACSCalibur flow cytometer (BD biosciences; software CellQuest Pro).

### Visualization of SOD1 monomers and dimers

Cells were rinsed once in PBS prior to lysis in lysis buffer (25 mM HEPES (PH 7.5), 150 mM NaCl, 1% NP-40, 100 mM MgCl_2_ 6H_2_O, 1 mM Na_3_VO_4_, 1 mM EDTA, 1 mM PMSF, and protease inhibitor cocktail). 5 mg/ml protein lysis was incubated for 30 min at room temperature with or without 20 mM Dimethyl pimelate. The samples were incubated with 0.2 M Tris-HCl (PH 7.5) for 15 min at room temperature and analyzed using immunoblotting.

### Clonogenic assay

Cells were plated in triplicate (500 cells/well in 6 well plates), treated with indicated doses of CPT and allowed to grow for 2 weeks. Plates were fixed in methanol and stained with 0.5% crystal violet solution. Colonies were counted and results were expressed as percentages of the untreated control.

### Animal studies

Animal studies using patient derived xenograft models were conducted by Crown Bioscience, Inc (Taicang, China) in strict accordance with the Guide for the Care and Use of Laboratory Animals of the National Institutes of Health. Prior to the initiation of treatment, mice were randomized into control and treated groups (*n* = 6 per group). Mice were treated intravenously with CPT-11 daily at 10 mg/kg or vehicle control for 21 consecutive days. Tumor growth was monitored by the measurement of tumor size using caliper every other day. Mice were sacrificed and tumor tissues were collected 2 hr after the last dosing.

### Statistical analysis

A two-tailed independent Student's t test was used to compare the continuous variables between the two groups. The *p* value < 0.05 was considered to be statistically signiﬁcant.

## SUPPLEMENTARY FIGURES AND TABLES


