# Supplementary material for: Acetylation at lysine 71 inactivates superoxide dismutase 1 and sensitizes cancer cells to genotoxic agents
Source: Oncotarget. 2015 May 4;6(24):20578–91. doi: 10.18632/oncotarget.3987 (PMC4653027; doi:10.18632/oncotarget.3987)
Supplement: Supplementary file 1 [file oncotarget-06-20578-s001.pdf]

Acetylation at lysine 71 inactivates superoxide dismutase 1 and sensitizes cancer cells to genotoxic agents

Supplementary Material

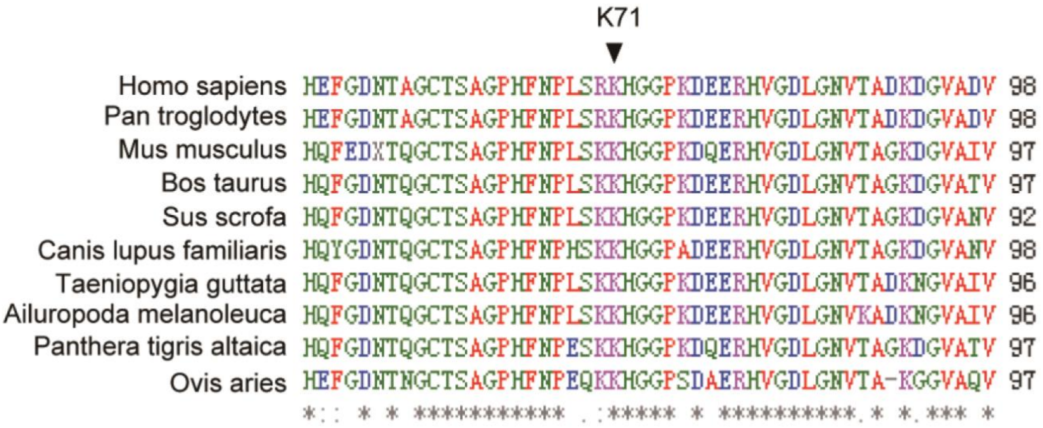

Figure S1: Alignment of SOD1 K71 across diverse species.

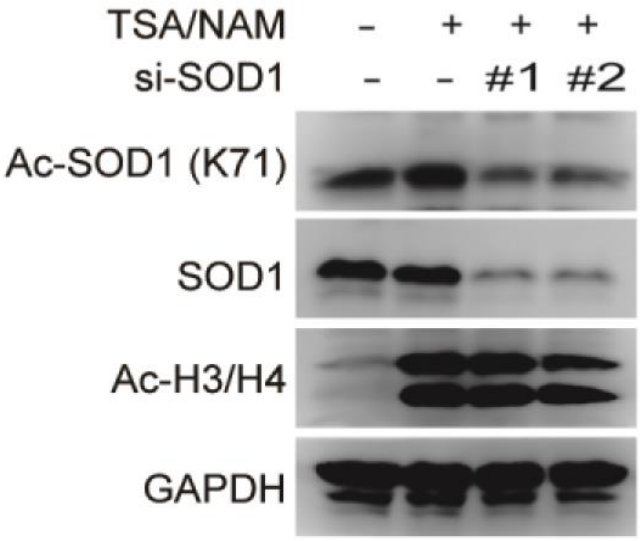

Figure S2: SOD1 siRNA knock down decreased ac-SOD1 level upon TSA/NAM treatment. HCT116 cells were transfected with scrambled or SOD1 siRNA (#1 or #2). After 48 h, cells were treated with TSA (500 nM) /NAM (10 mM) for 12hr. SOD1 K71 acetylation was detected by Ac-SOD1 (K71) antibody.

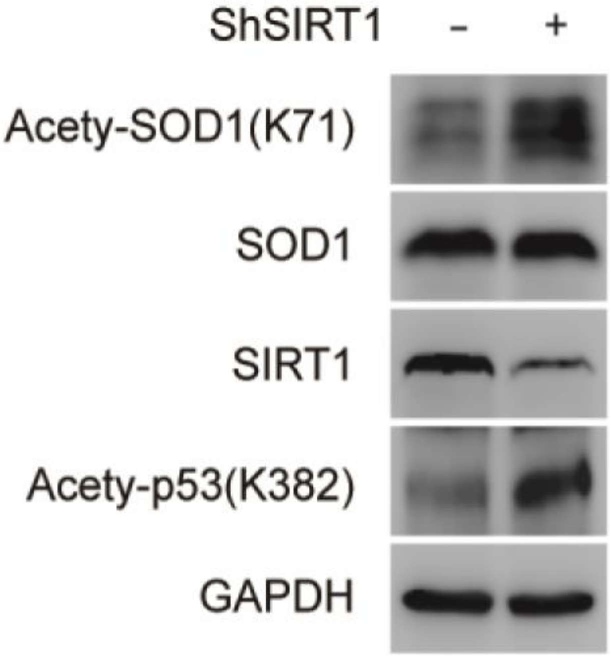

Figure S3: Stable knockdown of SIRT1 increases SOD1 acetylation. SIRT1 stably disrupted (shSIRT1) HCT-116 cells were transfected with Flag-tagged SOD1 and treated with NAM for 12 hr. SOD1 acetylation was detected by anti-Ac(K71) antibody..

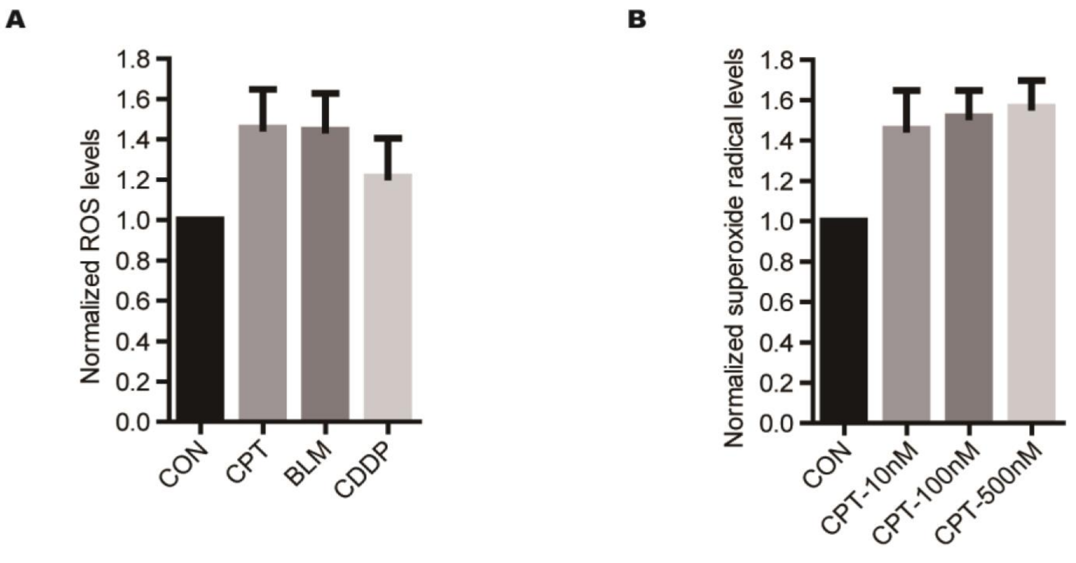

Figure S4: Genotoxic anticancer agents increase ROS generation. HCT116 cells were treated with **(A)** camptothecin (CPT, 100 nM), bleomycin (BLM, 10 μM) or cisplatin (CDDP, 20 μM) for 12 hr or **(B)** CPT at indicated concentrations for 12 hr. The cytosolic superoxide level was measured by DHE staining and FACS analysis. Mean ± SE (n = 3).

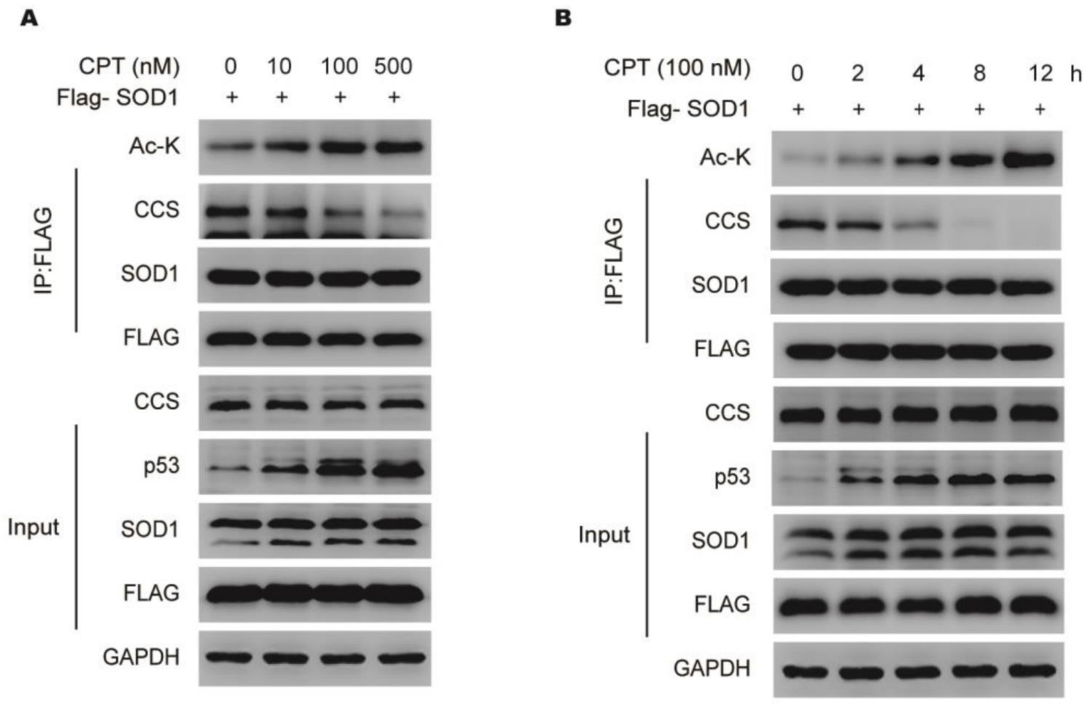

Figure S5: CPT treatment disrupts the interaction between SOD1 and CCS in a dose- and time-dependent manner. Flag-tagged SOD1 was transfected into HCT116 cells and then treated with **(A)** CPT at indicated concentrations for 12 hr or **(B)** 100 nM CPT for indicated time. Immunoprecipitation was performed using anti-Flag agarose. The interaction between SOD1 and CCS in the precipitated protein complex was assessed using immunoblotting.

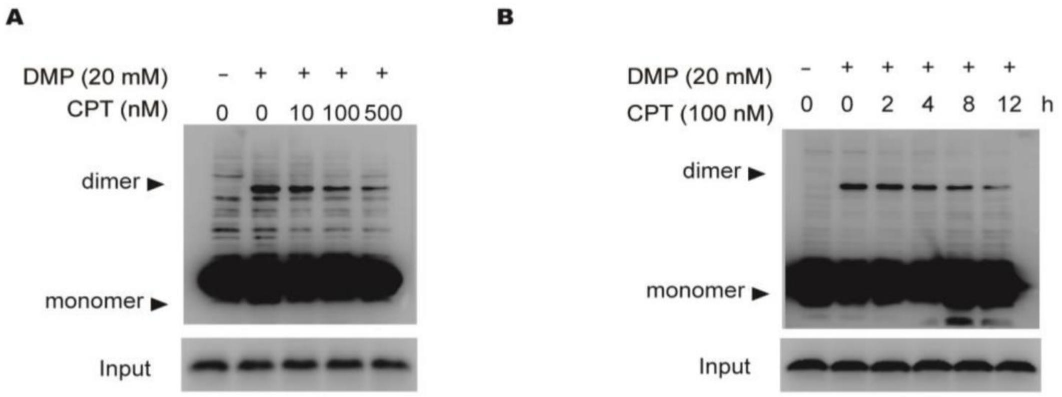

Figure S6: CPT treatment decreases SOD1 homodimers in a dose- and time-dependent manner. HCT116 cells transfected with flag-tagged SOD1 were treated with **(A)** CPT at indicated concentrations for 12 hr or **(B)** 100 nM CPT for indicated time. Cells lysis in the presence or absence of DMP (20 mM) were assessed by immunoblotting.

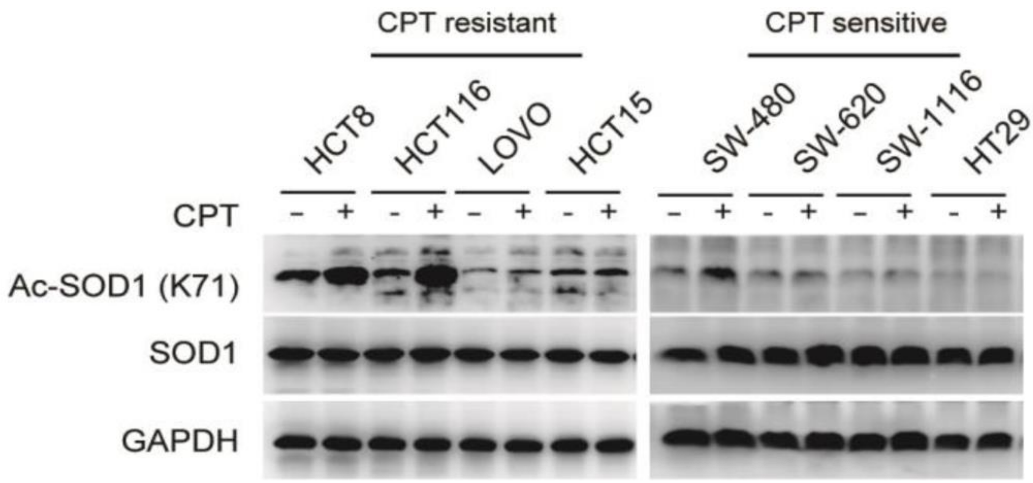

Figure S7: Ac-SOD1 level changes in responses to CPT treatment is not correlated with sensitivities of CPT treatment in colon cancer cells. Immunoblotting detection of SOD1 acetylation in CPT-sensitive and CPT-resistant colon cancer cells pretreated with DMSO or CPT (2 μM) for 12 hr. SOD1 K71 acetylation was analyzed by anti-Ac SOD1 (K71) antibody.

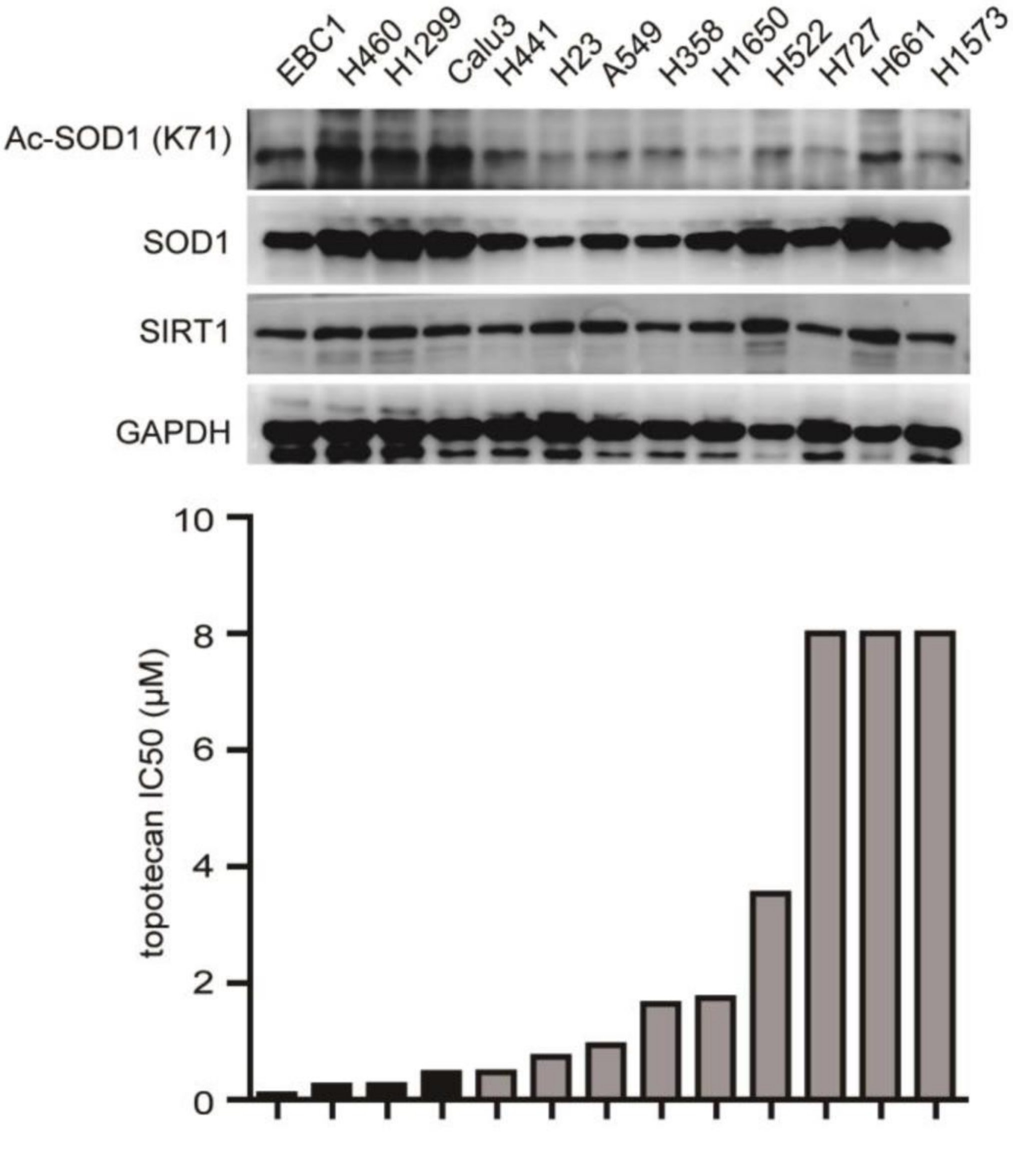

Figure S8: SOD1 acetylation is associated with the response to CPT treatment in lung cancer cells. Immunoblotting detection of SOD1 acetylation in CPT-sensitive and CPT-resistant lung cancer cell lines. SOD1 K71 acetylation was analyzed by anti-Ac SOD1 (K71) antibody.

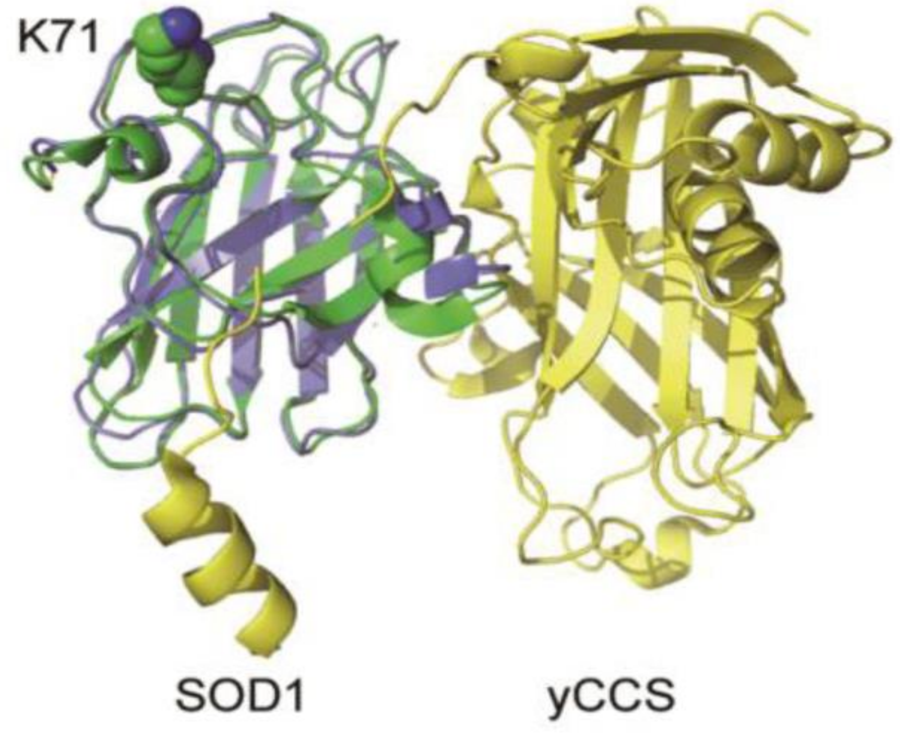

Figure S9: Alignment of human SOD1 structure to the crystal structure of ySOD1-yCCS complex. human SOD1(blue); ySOD1(green); yCCS(yellow).

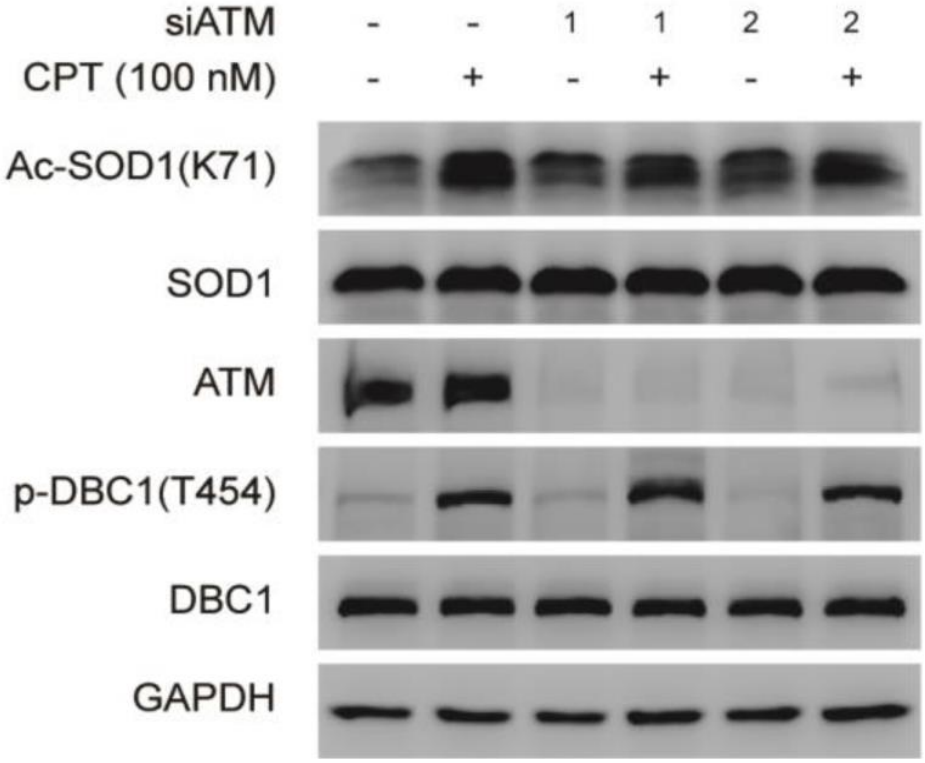

Figure S10: ATM/ATR-DBC1 signaling is not invovled in CPT induced SOD1 acetylation . HCT-116 cells were transfected with siATM and then exposed to CPT. Cell extracts were analyzed by immunoblotting.

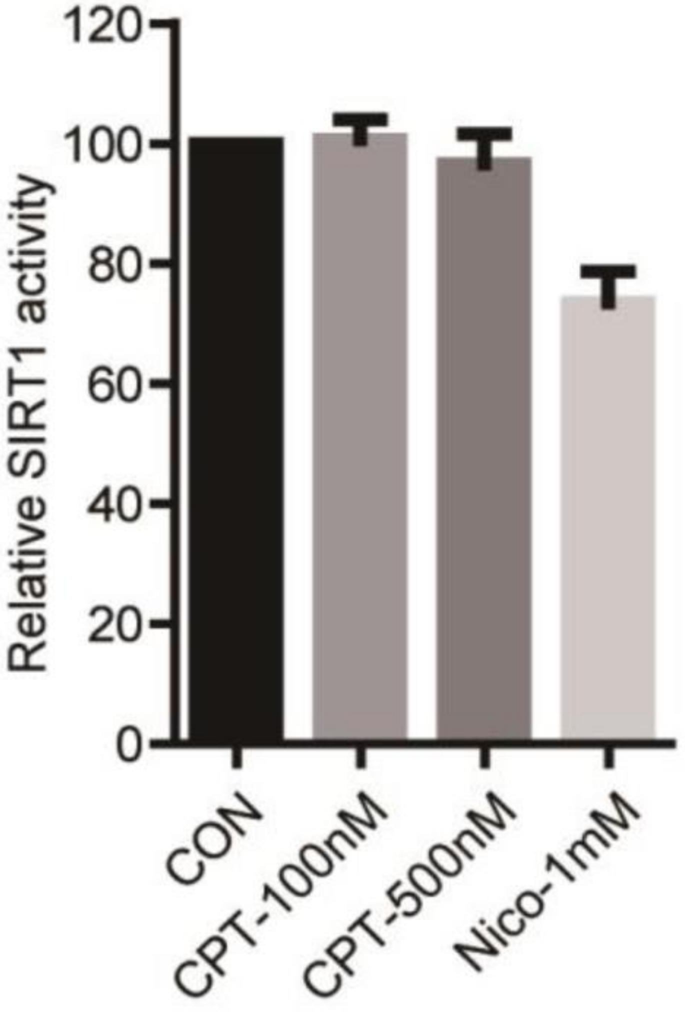

Figure S11: SIRT1 activity is not affected by CPT treatment in HCT116 cells. HCT116 cells were treated with CPT at indicated concentrations for 12 hr. SIRT1 activity in the cell extracts was analyzed using a SIRT1 activity detection kit (Sigma). Nicotinamide (NAM) was used as a postive control.
